# Belladonna in Poisoning by Opium

**Published:** 1880-01

**Authors:** 


### BELLADONNA IN POISONING BY OPIUM.

Dr. C. H. Lewis, in the November number of the Detroit *Lancet*, gives an interesting account of a case of poisoning with morphine, treated with subcutaneous injections of atropine. He believes belladonna to be the remedy *par excellence* in opium-nareosis, but that it must be given in heroic doses.

On the 22d of August, 1879, a lady took by mistake fully half a teaspoonful of morphia sulphate. About an hour was spent by another physician in procuring emesis, before Dr. Lewis saw the patient. At this time the stage of excitement was passing into that of stupor; the face was flushed, the eyes lustrous, but very prone to close, the pupil contracted, the skin warm and the pulse full and slightly accelerated. The patient could answer questions, but the last words would be lost in sleep, from which she would be suddenly roused by the head dropping forward.

The stomach was emptied with a full dose of sulphate of zinc, filled with water and emptied again, then a strong decoction of coffee given, at intervals, as long as the ability to drink it remained. Later, about two fluid drachms of the fluid extract of coffee were administered hypodermically. One twenty-fourth of a grain of sulphate of atropia was now injected subcutaneously, in fifteen minutes one-sixteenth of a grain, and the latter amount again in fifteen minutes.

No effect being perceived at the end of thirty minutes, one-eighth of a grain was injected. Not long after, the pupils began to dilate, and in an hour covered about one-half the iris. They remained at this degree of dilatation, and utterly unresponsive to light, with conjunctivæ insensible to touch. Notwithstanding this expansion of the pupils, and although derivatives to the skin were vigorously applied, the stupor became more profound, respiration more slow and shallow, the pulse more frequent and feeble, and the surface more cold and pale. Although none of the physicians present had any expectation of her recovery, the doctor continued to administer one-eighth of a grain, at intervals of about an hour. Faradization was employed for fully two hours, with the apparent result of raising the respiration from seven to twelve per minute. The first injection was given at 1 P. M., and the last at 7:15 P. M., and after the last dose its effect on the surface, pulse and respiration began slowly to manifest itself. At midnight the pulse was 100, regular and stronger, respiration 14, full and easy; face slightly flushed, and entire surface warm. Improvement progressed, and at 4 A. M. she fully awoke, the period of profound coma having been fourteen hours, and one grain and one-sixteenth of atropia having been administered subcutaneously.

